# Patient‐Centered Assessment of Palatal Donor Site Healing Using Platelet‐Rich Fibrin Versus Collagen: A Randomized Controlled Trial

**DOI:** 10.1155/ijod/3633056

**Published:** 2026-09-30

**Authors:** Manish Rathi, Randhir Singh, Saravanan Sampoornam Pape Reddy, Delfin Lovelina Francis

**Affiliations:** ^1^ Department of Periodontology Army Dental Centre (Research and Referral) New Delhi India; ^2^ Military Dental Centre Khadakwasla Pune India; ^3^ Saveetha Dental College and Hospitals Saveetha University Saveetha Institute of Medical and Technical Science Chennai Tamil Nadu India saveetha.com

## Abstract

**Introduction:**

Effective care of the palatal donor site after graft removal is vital for patient comfort and wound recovery. This study evaluated the effectiveness of platelet‐rich fibrin (PRF) compared with a collagen sponge as a dressing for palatal donor sites and its effects on patient‐reported outcome measures.

**Materials and Methods:**

Patients requiring soft‐tissue augmentation were enrolled in this randomized controlled clinical trial. They were divided into two groups: PRF (experimental group) and collagen sponge (control group). De‐epithelialized free gingival grafts were harvested from the palate, and donor sites were treated with either PRF or collagen sponge dressings. Patient‐reported outcomes and wound‐healing rates were evaluated.

**Results:**

Physical pain and physical disability scores were significantly better in the PRF group. Postoperative pain scores measured using the visual analog scale (VAS) were notably lower in the PRF group. No significant differences were found in psychological disability, social disability, or handicap scores between the groups.

**Conclusion:**

PRF appears to offer superior outcomes compared with collagen sponge for palatal donor‐site management, including better patient comfort, faster healing, and cost‐effectiveness. Further research is needed to establish the advantages of PRF over conventional treatments.

## 1. Introduction

A thin gingival phenotype around teeth and implants can induce significant dental problems, such as dentinal hypersensitivity and peri‐implant diseases, as well as esthetic concerns. If the keratinized gingival tissue is wider than 2 mm, oral hygiene can be effectively maintained [1]. Therefore, a lack of keratinized tissue coupled with a thin gingival phenotype may adversely affect tooth health. These soft‐tissue deformities can be corrected using autologous soft‐tissue grafts. Free gingival autografts and, more recently, de‐epithelialized free gingival grafts have been successfully used to enhance keratinized tissue zones [2].

However, gingival recession treated using subepithelial connective tissue grafts (CTGs) elicits more predictable outcomes. Both surgical procedures involve harvesting soft tissue, that is, epithelial and palatal CTGs, from the first premolar to the first molar region, resulting in the creation of an open surgical wound. Compared with conventional subepithelial CTGs, donor‐site morbidity is relatively higher with free gingival grafts owing to secondary‐intention healing, leading to increased postoperative discomfort. However, de‐epithelialized free gingival grafting is a highly predictable procedure because of the superior donor tissue quality [3].

Free gingival grafting is effective in increasing the keratinized tissue width. The most common site for obtaining autogenous soft‐tissue grafts is the palatal mucosa, specifically the area extending from the distal aspect of the canine root to the mesial palatal root of the first molar. However, free gingival grafts are limited by the need for two surgical sites and the risk of postoperative hemorrhage.

This process is widely used in periodontal treatments, and the palatal tissue is favored as it is a ready source of mesenchymal stem cells and possesses excellent self‐healing capabilities, relative accessibility, and qualities similar to those of the tissue surrounding the teeth. The donor palate site typically recovers within 2–4 weeks of graft harvest, although complete restoration may require an extended duration [4]. However, patients undergoing palatal harvesting experience severe postoperative discomfort, representing an unmet medical need. Moreover, other postoperative complications (e.g., hemorrhage, edema, additional discomfort, and delayed wound healing) are common [5].

Hyaluronic acid, platelet‐rich fibrin (PRF), and cyanoacrylate adhesives are effective biomaterials and biologics with the potential to reduce pain and accelerate healing [6, 7]. Furthermore, low‐level laser therapy and palatal stents induce beneficial effects on the wound healing rate after free gingival graft harvesting. To create standardized practices and precise standards for pain management, additional randomized clinical trials are required, which should consider confounding variables potentially affecting patient outcomes. To improve patient‐reported outcomes related to pain and discomfort during the recovery period after palatal graft harvesting, it is recommended to use a combination of the aforementioned treatments [8].

Common consequences of palatal tissue harvesting—frequently performed during periodontal plastic surgical procedures—are hemorrhage and edema, which can exacerbate pain. Bleeding and edema can delay wound healing by obstructing the tissues and increasing the risk of infection. The infection risk can be mitigated by careful intraoperative management; for example, precise incision methods and suitable hemostatic treatments, such as suturing and digital pressure application [9].

A recent clinical narrative review evaluated the effects of PRF on palatal wound healing in terms of patient‐reported outcome measures and found that PRF was superior to other treatments in reducing postoperative pain at the donor site in free gingival graft procedures [10]. This study also advocated more clinical trials with standardized protocols for PRF preparation and outcome measures to allow effective comparison and integration into clinical recommendations.

This randomized controlled trial (RCT) evaluated the effectiveness of using PRF compared to collagen for healing following donor tissue retrieval, with the aim of providing evidence‐based insights for improving palatal wound care in periodontal plastic surgery.

## 2. Materials And Methods

This study involved two groups: an experimental group receiving PRF and a control group receiving a collagen sponge. The study followed the CONSORT guidelines and adhered to the Declaration of Helsinki (updated in 2013). The Supporting Information 1: CONSORT flowchart and Supporting Information 2: CONSORT checklist are attached as supporting information files [11]. Ethics committee approval was obtained (number: 25CNAHMMD1203; date: March 12, 2024), and the trial was registered with CTRI (CTRI/2024/03/064205). Written informed consent was obtained from all study participants.

The inclusion criteria were: age 20–50 years; male or female; patients with multiple marginal tissue recessions requiring soft‐tissue augmentation using de‐FGG. Immunocompromised individuals and those with a history of irradiation treatment in the head and neck region, active periodontal disease, or bone metabolic disorders were excluded.

The required sample size was calculated based on a prior study that focused on the complete epithelialization of the palatal wound or donor site by the third week as the main outcome [12]. A minimum of 18 patients in each group was required to achieve 90% power at a 5% significance level. To account for dropouts, 20 patients were assigned to each group.

Initially, 72 patients were evaluated for eligibility, of whom 43 were selected based on the inclusion and exclusion criteria and other considerations from the Division of Periodontics at the Military Dental Centre, Khadakwasla. Therefore, a total of 43 participants were initially stratified by age and sex. After stratification, further randomization was carried out using a coin‐toss method to allocate participants to the experimental group (*n* = 21) or control group (*n* = 22). However, 3 participants withdrew from the study after initial inclusion because they were unwilling to undergo palatal tissue graft harvesting for the surgical procedure. The remaining 40 participants, with 20 in each group, received the allocated treatment.

The primary outcomes were patient‐reported outcome measures and wound healing outcomes of two surgical dressings applied to donor sites in the palatal region: PRF and collagen sponge. The patients and the outcome assessor for the wound healing index were blinded to the group assignment.

The initial phase of periodontal treatment was delivered in accordance with the S3 treatment guidelines, encompassing therapeutic steps 1 and 2, followed by maintenance therapy [13]. In the surgical phase (step 3), following successful administration of 2% lignocaine HCl with epinephrine (1:100,000), without sedation, a single surgeon (Manish Rathi) performed all surgical procedures.

In both groups, the de‐epithelialized free gingival graft procedure was performed following the previously described surgical technique [2]. All grafts were placed on a periosteal bed for root coverage and gingival phenotypic modification. A 10 × 10 × 1 mm^3^ graft was harvested from the palatal area, located ~2–3 mm apical to the marginal gingiva (Figures 1 and 2). The graft was securely placed at the recipient site using 5–0 monofilament polypropylene sutures. In both groups, venous blood (10 mL) was collected and placed in a centrifuge (REMI R‐4C Model R‐43; fixed‐angle rotor). To achieve an effective g‐force of 400 × *g* for L‐PRF preparation, centrifugation was carried out at 2700 rpm for 12 min in sterile glass test tubes, and the resultant PRF clot was removed [14]. The PRF was compressed against the recipient site in both groups. In the control group, the palatal donor site received collagen sponge dressings stabilized with 5–0 expanded polytetrafluoroethylene sutures (Figure 3A,B). In the experimental group, PRF was also used on the palatal donor site and stabilized using 4–0 expanded polytetrafluoroethylene crisscross sutures (Figure 4A,B).

**Figure 1:**
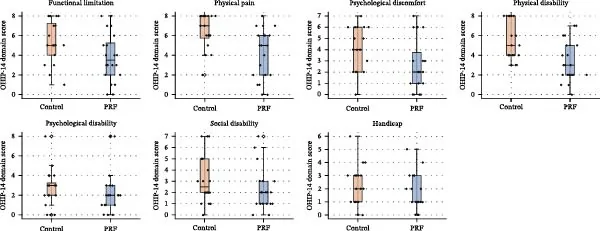
Changes in OHIP‐14 total scores from baseline to the 4‐week postoperative follow‐up, indicating an improvement in oral health‐related quality of life.

**Figure 2:**
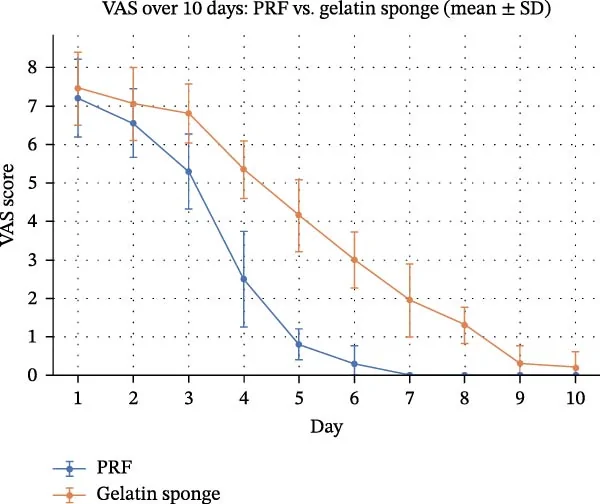
Visual representation of VAS pain scores at baseline and on Days 1–7, 10, 14, and 28. Bars indicate mean values; whiskers represent minimum and maximum values. Individual points beyond the whiskers represent statistical outliers, as defined by 1.5 times the interquartile range. VAS, visual analog scale.

**Figure 3:**
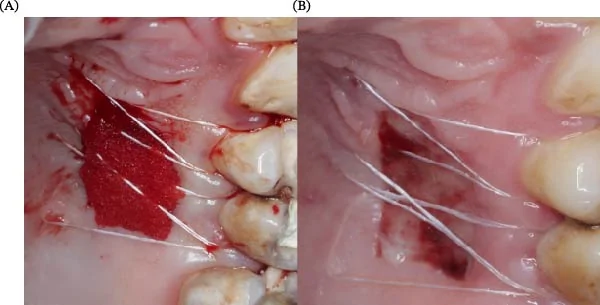
Procedural outcomes of the control group (collagen dressing). (A) Postoperative. (B) 1‐week postoperative.

**Figure 4:**
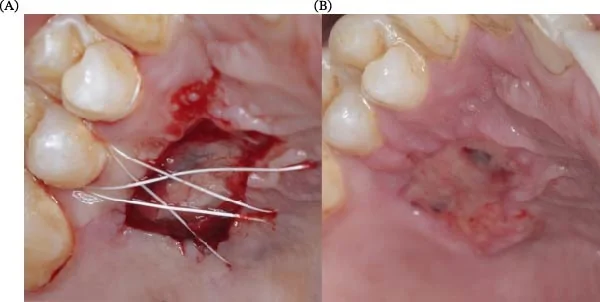
Procedural outcomes of the experimental group (platelet‐rich fibrin). (A) Postoperative. (B) 1‐week postoperative.

To ensure homogeneity, a similar suture material was used postoperatively in both groups. Patients were prescribed 400 mg ibuprofen three times daily for 3 days. Similar postoperative instructions regarding oral hygiene maintenance were given to patients in both groups. Postoperative assessment was carried out at 1 week, and sutures were removed from both the donor and recipient sites; each site was then flushed with normal saline. A second postoperative assessment was carried out at 2 weeks.

### 2.1. Patient‐Reported Outcomes

The Oral Health Impact Profile (OHIP‐14) was used to evaluate patients’ quality of life related to oral health [15]. The OHIP‐14 includes 14 questions covering 7 domains:

i. Functional limitation

ii. Physical pain

iii. Psychological distress

iv. Physical disability

v. Psychological disability

vi. Social disability

vii. Handicap.

Each domain is rated on a 5‐point Likert scale ranging from 0 (never) to 4 (very often), with a total possible score of 56 and higher scores indicating a poorer quality of life. Patients rated their pain intensity and discomfort on a 10‐cm visual analog scale (VAS), where 0 signifies “no pain” and 10 denotes the “worst pain possible.” The level of pain they experienced from the day before to 7 days following the surgery was recorded.

### 2.2. Wound Healing Assessment

Donor‐site wound healing was evaluated using a clinical wound‐healing score modified from Landry et al. [16], which included tissue color, bleeding on palpation, granulation tissue, and epithelialization. Healing was scored as follows: 1 = very poor, 2 = poor, 3 = good, 4 = very good, and 5 = excellent. Because wound‐healing assessment was required at 4 weeks postoperatively, an independent, calibrated, blinded examiner assessed wound healing and recorded the scores.

## 3. Statistical Analyses

All statistical analyses were performed using a repeated‐measures framework to account for the correlated structure of OHIP‐14 dimension scores within each participant across seven OHIP‐14 domains: functional limitation, physical pain, psychological discomfort, physical disability, psychological disability, social disability, and handicap. For each participant, domain‐level scores were computed by aggregating item‐level responses within the corresponding OHIP‐14 dimension as structured in the dataset. Two independent groups were analyzed: PRF (case group, *n* = 20) and collagen sponge (control group, *n* = 20). A linear mixed‐effects model (LMM) was employed to evaluate differences between groups while accounting for repeated measurements across domains within subjects. The model specification included the following: fixed effects: treatment group (PRF vs. collagen sponge), OHIP‐14 domain (7 levels), and their interaction; random effects: subject‐specific effects for intraindividual correlation.

The dependent variable was the OHIP‐14 domain score. To estimate treatment effects within each domain, post hoc analyses were derived from the fitted mixed model, comparing the PRF and control groups across all seven domains. To control for type I error inflation due to multiple comparisons, the Holm–Bonferroni correction was applied across the seven domain‐specific tests. Adjusted *p*‐values (Holm *p*‐values) were reported, and statistical significance was set at *p* < 0.05. All statistical tests were two‐tailed. Results are presented as mean differences (PRF − control) with 95% confidence intervals (CIs).

## 4. Results

Patient sex and age distributions, mean daily VAS scores, and OHIP‐14 scores collected on Days 1–7, 10, 14, and 28 are presented in Table 1. The PRF group exhibited significantly lower scores than the collagen sponge group for physical pain (mean difference = −2.30; 95% CI: −3.55 to −1.05; Holm‐adjusted *p* = 0.002) and physical disability (mean difference = −2.10; 95% CI: −3.35 to −0.85; Holm‐adjusted *p* = 0.006). No significant differences were detected in psychosocial domains after correction, suggesting that although PRF improves physical outcomes, its impact on psychological and social aspects may be limited or may require larger sample sizes to detect.

**Table 1:** Age and gender distribution of study participants.

Title	Site A (collagen) (*n* = 20)	Site B (PRF) (*n* = 20)	Total
Age (years)			
18–29	03	04	07
30–39	11	12	23
40–49	06	04	10
Gender			
Male	12	11	23
Female	08	09	17

The repeated‐measures analysis demonstrated a highly significant group × day interaction (*χ* ^2^(9) = 640.998, *p* < 0.001), indicating that the pattern of change in VAS scores over time differed significantly between the PRF and collagen sponge groups. A significant main effect of time was also observed (*χ* ^2^(9) = 2954.207, *p* < 0.001), reflecting a progressive reduction in pain scores across the 10‐day period in both groups. Post hoc day‐wise comparisons, adjusted for multiple testing using the Holm–Bonferroni method, revealed that the PRF group exhibited significantly lower VAS scores than the control group from Day 3 through Day 9 (*p* < 0.05 for all comparisons after adjustment). The greatest differences were observed between Days 4 and 6, indicating a more rapid reduction in pain in the PRF group during the early postoperative period (Figure 2). In contrast, no significant differences were observed on Day 1 and Day 2, and although a reduction in VAS was noted on Day 10 in the PRF group, this difference did not remain statistically significant after adjustment (*p* > 0.05). Figure 5 shows the comparison of wound healing scores at 4 weeks postoperatively according to the Landry, Turnbull, and Howley Index (LWHI) assessment [16] (Table 2).

**Figure 5:**
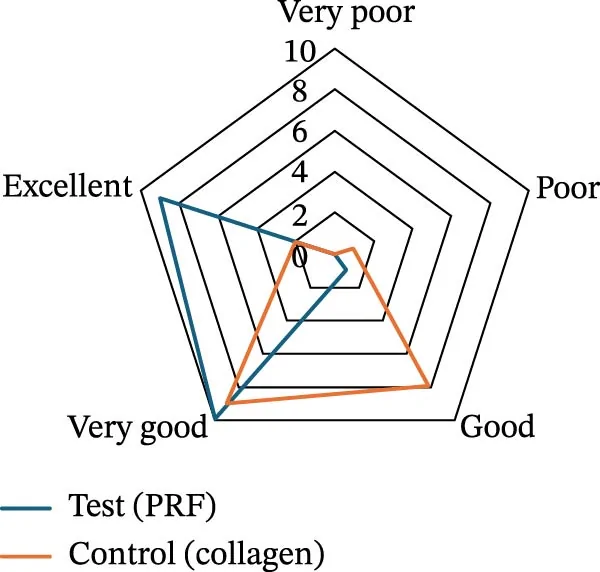
Landry, Turnbull, and Howley index (LWHI) assessment at 4 weeks postoperatively.

**Table 2:** Intergroup comparisons for OHIP‐14 domain.

OHIP‐14 domain	PRF (Mean ± SD) (*n* = 20)	Collagen sponge (Mean ± SD) (*n* = 20)	Mean difference (PRF − control)	95% confidence interval	Holm‐adjusted *p*‐value
Functional limitation	3.80 ± 2.48	5.30 ± 2.15	−1.50	−2.75 to −0.25	0.092
Physical pain	4.15 ± 2.56	6.45 ± 1.82	−2.30	−3.55 to −1.05	**0.002 ^∗^**
Psychological discomfort	2.65 ± 2.32	3.75 ± 2.10	−1.10	−2.35 to 0.15	0.335
Physical disability	3.35 ± 2.06	5.45 ± 1.93	−2.10	−3.35 to −0.85	**0.006 ^∗^**
Psychological disability	2.30 ± 2.23	2.70 ± 1.87	−0.40	−1.65 to 0.85	0.958
Social disability	2.30 ± 1.87	3.30 ± 2.27	−1.00	−2.25 to 0.25	0.348
Handicap	1.75 ± 1.37	2.20 ± 1.47	−0.45	−1.70 to 0.80	0.958

*Note:* Intergroup comparisons performed using linear mixed‐effects repeated‐measures model with subject as random effect and domain as within‐subject factor. Holm–Bonferroni correction applied to adjust for multiple comparisons across the seven domains. Negative values indicate lower OHIP‐14 scores (better outcomes) in PRF group. Bold values indicate statistically significant after correction.

^∗^Statistically significant at *p* < 0.05 after correction.

## 5. Discussion

Several treatments aimed at enhancing wound healing, reducing pain, and managing postoperative bleeding at the palatal donor site are currently available. Absorbable collagen sponges support hemostasis and offer a tissue regeneration scaffold, whereas cyanoacrylate tissue adhesives are used for wound closure to prevent bleeding, thereby accelerating healing [17, 18]. Oxidized regenerated cellulose is another hemostatic agent that promotes wound healing [19]. Hyaluronic acid, renowned for its moisturizing characteristics, promotes tissue regeneration and reduces inflammation [20]. Low‐level laser therapy stimulates tissue repair and pain relief, whereas ozone therapy improves wound healing by increasing oxygen delivery to the tissues [13, 21]. Moreover, compared to conventional treatments, PRF accelerates palatal wound healing and reduces pain and discomfort.

Supporting studies have reported that PRF promotes faster complete wound epithelialization and greater wound reduction compared with control treatments [22, 23]. Additionally, most studies have reported lower postoperative pain with PRF [4]. Notably, the present study measured patient‐reported outcomes using tools such as the OHIP‐14.

In a previous study evaluating PRF, the initial healing outcomes were similar to or slightly better than those of collagen dressings. Moreover, PRF is easier to handle and suture and is more economical than commercial collagen products [24]. Another study reported that PRF exhibited a better barrier function against bacterial invasion over 48 h compared with two collagen membranes [12]. Nevertheless, some studies have presented conflicting results: one study found no significant differences in early palatal wound healing, patient pain perception, or analgesic consumption between patients treated with PRF and those treated with a hemostatic agent as donor‐site dressings [25]. In the current study, although analgesic consumption was standardized across both groups, postoperative VAS scores were significantly different between the groups from Day 3 through Day 9, with the greatest differences observed from Day 4 to Day 6, suggesting reduced pain in the PRF group during the early postoperative period. However, it is noteworthy that by Day 10, the difference between groups was no longer statistically significant after correction, indicating that pain levels in both groups converged as healing progressed. This suggests that the benefit of PRF is most pronounced during the transitional healing period rather than at the final stage, when tissue repair is largely complete.

PRF promotes faster and more effective neovascularization and cicatricial tissue remodeling, leading to accelerated wound closure [26]. The sustained release of growth factors, such as vascular endothelial growth factor and platelet‐derived growth factor, from PRF enhances granulation tissue formation, collagen deposition, and angiogenesis [27]. Additionally, PRF stimulates fibroblast proliferation and collagen biosynthesis more effectively than recombinant growth factors alone [28].

One recent study focusing on patient‐reported outcomes documented significantly reduced psychological stress in patients receiving PRF compared with those receiving a hemostatic agent. However, the study parameters were recorded only 1 week postoperatively [29]. A comparative study evaluating the efficacy of PRF membranes in conjunction with allogenic bone grafts versus collagen membranes in the treatment of infrabony defects demonstrated overall satisfactory outcomes, favoring the PRF group [30]. Another important aspect is the type of tubes used for preparing PRF, which affects clot quality and growth factor release; additive‐free glass tubes produce a more natural fibrin matrix and better regenerative potential than silica‐coated tubes, which may alter the clot structure and introduce contaminants [31].

The present study focused on seven different aspects of patient‐reported outcomes. Notably, physical pain and physical disability differed significantly between the experimental and control groups. A similar study comparing the outcomes of PRF and collagen as palatal dressings concluded that PRF produced better responses in terms of patient morbidity, whereas other parameters were comparable between groups [19]. The current study extends these findings by providing a longitudinal, repeated‐measures analysis with appropriate statistical correction, thereby strengthening the evidence that PRF offers a sustained and clinically meaningful benefit over conventional materials such as collagen sponge. It primarily concentrated on evaluating patients’ responses to the procedure, which can be quantified in terms of patient outcomes. To our knowledge, this is the first study to document patient‐reported outcomes at different postoperative time points while monitoring outcome improvement over a 4‐week period. Importantly, the use of Holm–Bonferroni correction ensured that the observed significant differences were robust and not due to an inflated type I error from multiple comparisons (Table 3).

**Table 3:** Comparison of the healing scores obtained according to Landry, Turnbull, and Howley index (LWHI assessment) after 04 weeks.

Score	Site A frequency (%)	Site B frequency (%)
1. Very poor	0	0
2. Poor	1	0
3. Good	8	1
4. Very good	9	10
5. Excellent	2	9

Certain limitations of this study should be acknowledged. Because this study did not employ a split‐mouth design, interindividual variability in the treatment response is possible. Nevertheless, all surgical procedures were conducted by the same operator [MR], thereby minimizing operator and performance bias. In addition, advanced protocols, such as the low‐speed centrifugation concept and the horizontal centrifugation method instead of fixed‐angle centrifugation, could have been used. These considerations should be addressed in future clinical trials.

In conclusion, although both PRF and collagen sponge can enhance palatal wound healing, PRF offers superior outcomes in terms of patient comfort, healing speed, and cost‐effectiveness. This study concludes that the use of platelet‐rich fibrin may offer benefits for palatal donor‐site management in terms of patient‐reported outcome measures. Future studies with larger sample sizes and longer follow‐up periods are required to validate these findings and explore the benefits of PRF in soft‐tissue grafting procedures.

## Author Contributions

Conceptualization, investigation: Manish Rathi and Randhir Singh. Formal analysis: Manish Rathi, Randhir Singh, and Saravanan Sampoornam Pape Reddy. Methodology: Manish Rathi and Randhir Singh. Writing – original draft: Manish Rathi. Writing – review and editing: Manish Rathi, Randhir Singh, Saravanan Sampoornam Pape Reddy, and Delfin Lovelina Francis.

## Funding

No funding was received for this manuscript.

## Disclosure

All authors agree to be accountable for the research presented.

## Conflicts of Interest

The authors declare no conflicts of interest.

## Supporting Information

Additional supporting information can be found online in the Supporting Information section.

## Supporting information


**Supporting Information 1** CONSORT Flowchart.

**Supporting Information 2** CONSORT Checklist.

## Data Availability

The data that support the findings of this study are available from the corresponding author upon reasonable request.
